# Leaf Litter Leachate Limits Fungal Pathogen Growth but Not Amphibian Infection

**DOI:** 10.1002/ece3.73668

**Published:** 2026-05-13

**Authors:** Emily L. Martin, Isabel Adarve‐Rengifo, Spencer R. Siddons, Paradyse E. Blackwood, Jessica Hua, Catherine L. Searle

**Affiliations:** ^1^ Department of Ecology and Evolutionary Biology University of California Irvine California USA; ^2^ Department of Biological Sciences Purdue University West Lafayette Indiana USA; ^3^ School of Applied Sciences and Engineering Universidad EAFIT Medellin Colombia; ^4^ Department of Zoology University of British Columbia Vancouver British Columbia Canada; ^5^ Hakai Institute Calvert Island British Columbia Canada; ^6^ Department of Forest and Wildlife Ecology University of Wisconsin–Madison Madison Wisconsin USA

**Keywords:** allochthonous inputs, amphibian disease, *Batrachochytrium dendrobatidis*, contaminants, leaf litter

## Abstract

Terrestrial leaf litter can release chemical compounds into the water, creating a solution called “leachate” that can have direct and indirect impacts on freshwater organisms. In this study, we investigated the direct effects of leaf litter leachates from six plant species on the fungal pathogen, *Batrachochytrium dendrobatidis* (Bd) in vitro, and their effects on tadpole infection in vivo, when tadpoles were reared in leachates before Bd exposure. In our in vitro experiment, all six types of leachates reduced the concentration of Bd zoosporangia, and there were mixed effects of leachate type on the concentration of Bd zoospores. Purple loosestrife leachate had the greatest effect on Bd in the in vitro experiment suggesting that this plant species may produce chemicals that are directly detrimental to Bd. The negative effects of leachates on Bd in vitro increased with leachate concentration. In our in vivo tadpole experiments, leachates had no measurable impact on the tadpole host survival, size, development, or infection with Bd. Our results indicate that leaf litter leachate can have direct negative effects on Bd, but these effects may not translate to large changes in infection in tadpoles. However, future studies are necessary to determine the comprehensive impact of leachates on aquatic amphibians and their pathogens in natural populations. These results highlight the importance of understanding terrestrial inputs into freshwater systems.

## Graphical abstract

Terrestrial leaf litter can have direct effects on aquatic organisms. We found that leaf litter leachates can have direct negative effects on a fungal amphibian pathogen (Batrachochytrium dendrobatidis), but these effects did not translate to large changes in infection.

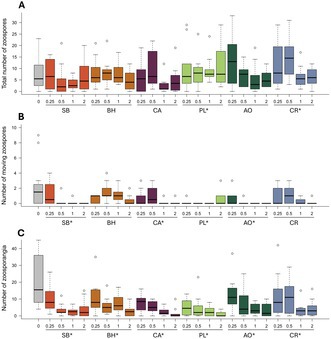

## 1 Introduction

Aquatic environments can be greatly impacted by terrestrial inputs (e.g., Likens and Bormann 1974; Häder and Barnes 2019; Sullivan and Manning 2019). One common source of inputs from terrestrial environments is the addition of leaves from terrestrial plants into freshwater environments (Fisher and Likens 1973; Drake et al. 2018). Plant leaf litter can alter freshwater systems by increasing habitat complexity, serving as a food source, and leaching compounds that shift water chemistry (reviewed in Stoler and Relyea 2020).

The concentration and composition of leaf litter in aquatic systems varies both temporally and spatially, and can have numerous effects on aquatic organisms. For example, depending on the plant species and the ecological context, the density of leaf litter in an aquatic system can be positively or negatively correlated with zooplankton abundance (e.g., Watkins et al. 2011; Cottingham and Narayan 2013; Fey et al. 2015). Leaf litter can alter aquatic species interactions including competition (DiGiacopo and Hua 2022), predator–prey interactions (Stoler and Relyea 2013; Burraco et al. 2018) and host‐pathogen interactions (DiGiacopo and Hua 2022). Some studies have found that leaf litter from invasive species has a larger detrimental effect on native aquatic organisms than litter from native plant species (Maerz et al. 2005; Burraco et al. 2018; Berta and Mott 2023). In all, the type (i.e., plant species) and concentration of leaf litter are important predictors of how aquatic systems are impacted.

When leaf litter interacts with water and releases secondary chemical compounds into aquatic systems, the solution that is created is known as “leachate” (Lush and Hynes 1973). Because these water‐soluble, chemical compounds vary greatly across plant species, the chemical composition of leachates can also vary depending on leaf litter type (Stoler and Relyea 2016; Stoler, Berven, and Raffel 2016). Not all leachates are equal in effect within aquatic systems; their composition of chemical traits (e.g., phenolic acids, dissolved carbon, nutrients) influences aquatic processes including productivity, water clarity and pH, decomposition, microbial activity, and consumer development and mortality (see examples in Stoler and Relyea 2020). For example, leaf‐litter derived nutrients can often be linked to variable primary productivity in these systems, while the quantity of phenolic acids may be more directly linked to aquatic species' mortality (Cohen et al. 2012; Stoler, Berven, and Raffel 2016; Stoler and Relyea 2020).

Leachates may also impact infectious disease in aquatic systems (Davidson et al. 2012; Stoler, Berven, and Raffel 2016), including the disease chytridiomycosis, which is caused by the amphibian fungal pathogen, *Batrachochytrium dendrobatidis* (hereafter “Bd”). This pathogen is associated with severe declines of amphibian populations across the world (reviewed in Fisher and Garner 2020). Within aquatic systems, there is large variation in the Bd infection prevalence and intensity across the landscape (e.g., Siddons et al. 2020; Zumbado‐Ulate et al. 2021) which could be explained, in part, by variation in leaf litter inputs. A previous study by Stoler, Berven, and Raffel (2016) found that increased leachate concentrations generally decreased Bd densities in vitro, but the effects of leachate varied by plant species and were greatest in leachates from plants with high phenolic content. Another study found that extract from *Eucalyptus camaldulensis* leaves reduced Bd density in vitro and the presence of leaves from these species reduced Bd infection in salamanders (Davidson et al. 2012). Thus, leachates can have direct negative impacts on Bd.

Amphibian hosts can also experience direct negative effects from plant leachates (e.g., Maerz et al. 2005; Watling et al. 2010; Earl and Semlitsch 2014; Burraco et al. 2018). For example, extract from the invasive and widespread purple loosestrife ( *Lythrum salicaria* ) increases mortality and slows development of American toad tadpoles ( *Anaxyrus americanus* ; Maerz et al. 2005). Reduced survival in tadpoles can occur with increased tannins and terpene concentrations (Earl et al. 2012). These negative effects on amphibians can be associated with the chemical composition of leachates. For example, the toxicity of secondary plant compounds from the invasive Eurasian watermilfoil ( *Myriophyllum spicatum* ), which is invasive in North America, was associated with reduced body size of northern leopard frogs ( *Lithobates pipiens* ; Curtis and Bidart 2017). However, there remains a lack of understanding of the impact of leachates on both aquatic amphibian hosts and Bd, particularly regarding how these effects ultimately impact infection outcomes.

In this study, we aimed to (1) quantify the direct effects of native and invasive leachate types (i.e., plant species used to create leachate) and concentrations on Bd in vitro, and (2) test the effects of leachate type on Bd infection in tadpoles via changes to the host before Bd exposure (in vivo). We predicted that (1) leachates would directly inhibit the growth of Bd in vitro, and (2) pre‐exposure to leachates would alter tadpole susceptible to Bd infection. By testing leachates from three native and three invasive species in the United States (see details in Materials and Methods), we can determine if plant species origin influences the effect of leachates. By combining results from two experiments, we can help develop an understanding of the impacts of leachates on Bd infection in tadpoles.

## 2 Materials and Methods

### 2.1 Preparation of Leaf Litter Leachates and Bd Cultures

For all experiments, we created different leachate types using leaf litter from six plant species acquired from Binghamton University Nature Preserve, New York, USA (42.08°N, 75.97°W). The plant species consisted of three species that are native to North America (steeplebush; *Spiraea tomentosa* , black huckleberry; *Ericaceae* sp., and cattail; *Typhaceae* sp.) and three species that are invasive in this region (purple loosestrife; *Lythrum salicaria* , autumn olive; *Elaeagnus umbellate*, and common reed; *Phragmites australis* ). All these species can commonly be found in or near wetlands (Blossey et al. 2001; Thompson and Jones 2001; Bellavance and Brisson 2010; USDA 2022). Throughout the experiments, we used unchlorinated well water unless otherwise stated. Dry leaves from each species were individually soaked for 40 days in water at a concentration of 10 g L^−1^ for the in vitro experiment and 1 g L^−1^ for the in vivo experiment in indoor 50 L cattle tanks following methods by DiGiacopo et al. (2019) (Figure S1). During the soaking process, we stirred and fully submerged the leaves every 3–4 days and topped off tanks with water to compensate for evaporation. Additionally, we created a control treatment of water with no leaf litter treated in the same manner. After the end of the 40‐day soaking period, we strained the leachates through a 153 μm filter mesh to remove leaf particulates and then sterilized them with UV lights.

For all experiments, we used a strain of Bd isolated from a leopard frog (*Lithobates* sp.) in Ohio, USA (strain JSOH1) maintained in 1% tryptone liquid broth. To grow Bd for the experiments, we added 2 mL of liquid culture broth to 1% tryptone agar Petri dishes and incubated them at 21°C for 7 days for the in vitro experiment and 10 days for the in vivo experiment. Bd is transmitted via a flagellated zoospore stage which then encysts to develop into a zoosporangium (a spore case in which zoospores develop) that asexually produces more zoospores (Longcore et al. 1999). To harvest the Bd from the Petri dishes, we flooded dishes with 3 mL of autoclaved RO water for 15 min to allow zoospores to release into the water. We then pooled the solutions across dishes and quantified zoospore concentrations using a hemocytometer.

### 2.2 In Vitro Experiment: Direct Effects of Leachates on Bd

We first tested the direct effects of leachates on Bd by culturing the pathogen in vitro in different leachate types and concentrations. This experiment had a 4 × 6 factorial design with six leachate types (described above) and four concentrations of each leachate (0.25, 0.50, 1.00 and 2.00 g L^−1^). We created the different leachate concentrations via dilutions with water from a starting concentration of 10 g L^−1^. We also included an additional, no‐leachate control (well water) for a total of 25 treatments, each replicated 12 times. We then added 1 g L^−1^ tryptone powder to each leachate treatment to encourage Bd growth and the solutions were autoclaved before use in the experiment. Experimental units were 1.5 mL microcentrifuge tubes with 900 μL of leachate + tryptone solution and 100 μL Bd solution created by pooling water from eight Bd‐inoculated Petri dishes into a single beaker and diluting the solution to a concentration of 5 × 10^5^ zoospores mL^−1^ with water, so each experimental unit received 5 × 10^4^ zoospores.

We incubated the solutions at room temperature (20°C–21°C) in the dark (i.e., placed into an insulated container that was sealed and stored in an enclosed space). Starting 10 days after the initial setup, we used a hemocytometer to quantify the concentration of total zoospores (moving and unmoving), moving zoospores, and zoosporangia in 0.1 μL of the liquid solution from each replicate. We classified zoospores based on the presence of a flagellum, while zoosporangium were identified based on the presence of rhizoids (Longcore et al. 1999). We also looked for zoospores and zoosporangia in control tubes without Bd, and we did not see any signs of Bd, confirming that our quantification methods were in fact measuring Bd. Due to the large number of replicates, we quantified zoospores and zoosporangia over 4 days so that the incubation period for this experiment was 10–13 days, with replicates measured in a random order.

### 2.3 Amphibian Collection and Tadpole Husbandry

Our host species for the in vivo experiment was the American bullfrog ( *Lithobates catesbeianus* ; hereafter, “bullfrog”), a common species in the eastern USA, that is susceptible to Bd infection. Bullfrogs are considered relatively tolerant of Bd infection, especially at the tadpole stage, and sustain high infection intensity (Garner et al. 2006; Schloegel et al. 2010; but see Gervasi et al. 2013; Eskew et al. 2015). Tadpoles of bullfrogs and most amphibian species can become infected with Bd without experiencing mortality from Bd infection (e.g., Searle et al. 2011). We collected three egg masses of bullfrog from Purdue Wildlife Area, a permanent wetland located in West Lafayette, Indiana, USA (40.45°N, 87.05°W). Eggs cannot be infected with Bd as they do not contain exposed keratin for Bd to infect (Marantelli et al. 2004). We reared eggs from each mass separately in 50‐gal indoor tanks filled with water, equipped with an aquarium air pump held at 21°C with a natural photoperiod created via a large window. Upon hatching, tadpoles remained in indoor tanks and were fed *ad libitum* with a mixture of fish flakes (Tetramin brand) and alfalfa pellets with water changes every 5 days. We used tadpoles at Gosner stage 25 (Gosner 1960) for the experiments; equal numbers were taken from the three original egg masses and randomly distributed among the experimental treatments.

### 2.4 In Vivo Experiment: Leachate Exposure on Tadpoles Before Infection

In the in vivo experiment, we exposed tadpoles to leachate before Bd exposure to isolate the impacts of leachates on infection via changes to the host. We had seven treatments with six leachate treatments (one for each leaf litter type) at a concentration of 1 g L^−1^, plus a well‐water control. Each treatment was replicated 18–20 times (Table S1). Tadpoles were held in 300 mL of their randomly assigned leachate treatment in 400 mL beakers for 20 days prior to Bd exposure. We then placed tadpoles into clean beakers with 300 mL well water and added Bd to create a concentration of 1.3 × 10^4^ zoospores in the beaker. The tadpoles remained in these beakers with Bd for an additional 5 days before the experiment ended. We also conducted an additional in vivo experiment with no well‐water control where tadpoles were exposed to leachates before, during, and after Bd exposure (Figures S2 and S3A; Tables S1 and S2).

We randomly assigned beakers spatially to one of 4 shelves in the laboratory. Tadpoles were checked daily for mortality. On the final day of the experiment, we euthanized individuals in an overdose of MS‐222 and preserved all individuals in ≥ 90% ethanol until processing for infection analysis. After euthanizing, we weighed and staged all animals (Gosner 1960).

We used quantitative PCR to determine the Bd infection status (infected or uninfected) and infection intensity (number of zoospores) of each tadpole that survived until the end of the experiment. We removed mouthparts (e.g., three teeth rows and keratinized jaws of a tadpole) of each tadpole using sterile dissecting scissors or a scalpel. We extracted DNA from each mouthpart using a Qiagen DNeasy Blood and Tissue Kit. Quantitative PCR was conducted via the “fast” method (Boyle et al. 2004; Kerby et al. 2013) with all mouthpart samples run in duplicate and Bd standards in triplicate ranging from 10^0^ to 10^3^. We considered a sample to be Bd‐positive if both duplicate wells were positive for Bd, and a sample to be Bd‐negative if only one or no wells amplified. This is a fairly conservative method for determining infection status and can lead to false‐negative results. We calculated infection intensity for each Bd‐positive individual as the mean of the two replicated raw quantities from the quantitative PCR output.

### 2.5 Statistical Analysis

All statistical analyses were performed in R Version 4.5.0 (R Core Team 2025). For the in vitro experiment, we constructed a generalized mixed effects model with a Poisson distribution for each response variable (total zoospores, moving zoospores, and zoosporangia; “glmer” function in the “lme4” package; Bates et al. 2015). We chose to use a Poisson distribution because these response variables are integers. Our fixed effects were leachate type (i.e., plant species), leachate concentration, and the interaction between leachate type and concentration, with the control treatment (clean water, concentration of zero) as the reference group. Our random effect was the day of data collection. The interaction between leachate type and concentration was non‐significant and was therefore removed from the final models to improve parsimony.

For the in vivo experiment, we used leachate type as the predictor for all models (*n* = 7 levels). To compare infection prevalence and intensity across treatments, we only used information from tadpoles that survived the entire experiment, because animals that died early had less time for infection to develop (Table S1). To compare proportion infected across leachate types, we used a binomial generalized linear model. We compared infection intensity across leachate types using a Kruskal–Wallis test due to violation of assumptions required for parametric analysis; significant effects were followed with a Dunn's test (“dunnTest” function in “FSA” package; Ogle et al. 2025) with adjusted *p*‐values based on the Holm method. We used a Cox proportional hazards model to evaluate the effect of leachate type on tadpole survival (“coxph” function in the “survival” package; Therneau 2024). We compared ending mass across treatments using a Kruskal‐Wallis test and stage using a Poisson generalized linear model. When possible, in addition to results from the statistical tests, we also report untransformed β estimates and SE of effect size from our models, which include overall effects of each treatment.

## 3 Results

### 3.1 In Vitro Experiment: Direct Effects of Leachates on Bd

**FIGURE 1:**
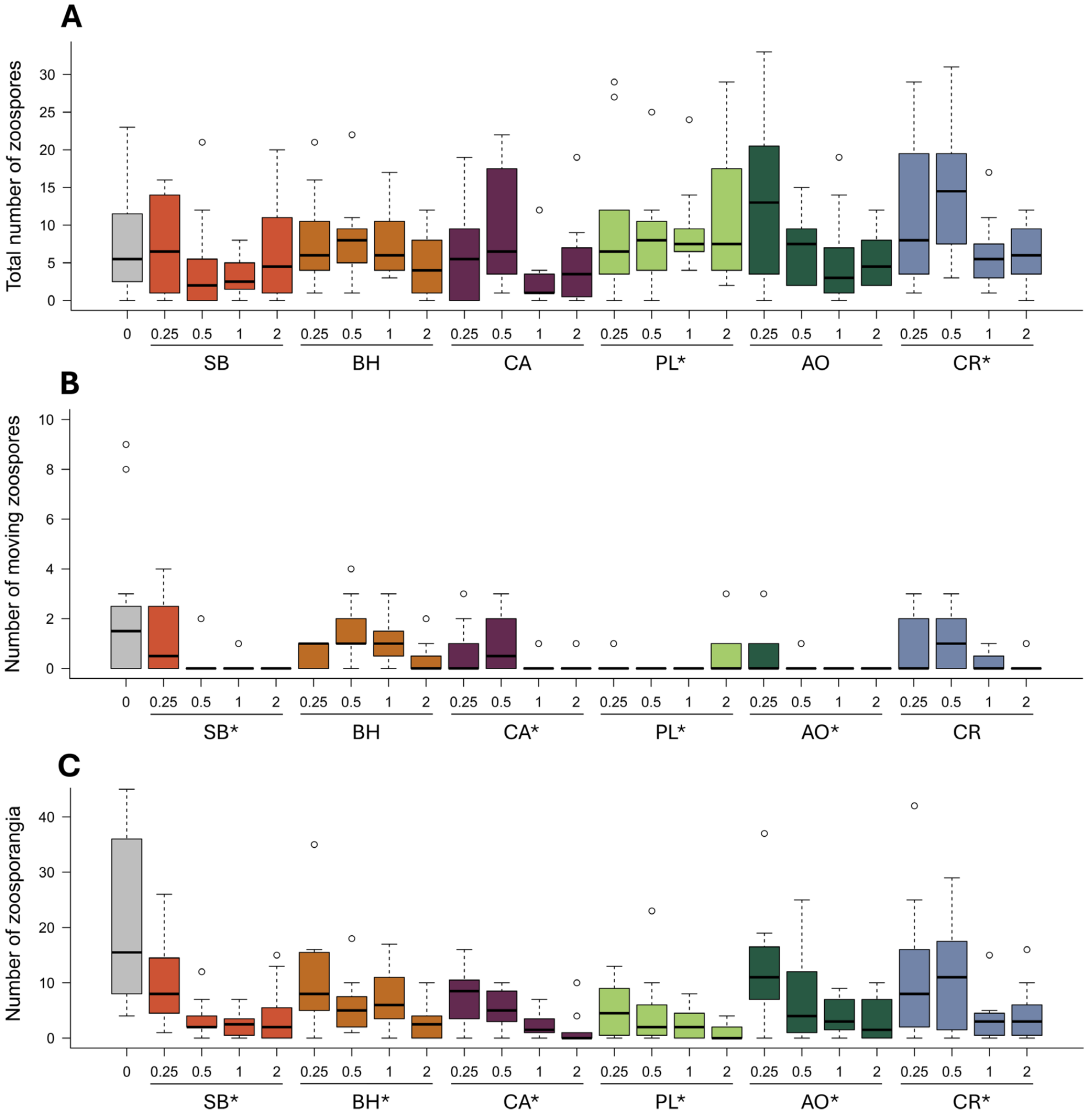
Results of the in vitro experiment: Direct effects of leachates on Bd. Results show the number of (A) total zoospores (moving and unmoving combined), (B) moving zoospores, or (C) zoosporangia that were found in the 0.1 μL sample from each replicate. The leachate types are represented as the native species SB (steeplebush), BH (black huckleberry), and CA (cattail), and the invasive species PL (purple loosestrife), AO (autumn olive), and CR (common reed). Leachate types indicated with an asterisk are those that differed significantly from the control treatment (left‐most treatment in gray). Concentrations of each leachate type are shown on the x‐axis in g L^−1^. Boxplots show median (center line) and interquartile range (box) for each treatment with circles representing outliers. There is one very high value of moving zoospores (B) that is not shown; it was in the cattail treatment with 0.5 g L^−1^ concentration and had 22 moving zoospores.

Increasing the leachate concentration decreased the total number of zoospores counted (*X* ^2^(1) = 50.66, *p* < 0.001, *β* = −0.235 ± 0.034; Figure 1A). Leachate type also impacted total zoospore counts (*X* ^2^(6) = 95.58, *p* < 0.001); zoospore counts were higher when grown in leachate from the invasive purple loosestrife (*β* = 0.299 ± 0.123, *p* = 0.015) and common reed (*β* = 0.326 ± 0.122, *p* = 0.008) compared to the control treatment (Figure 1A). There was also a nearly significant reduction in zoospore counts in the native steeplebush treatment compared to the control (*β* = −0.251 ± 0.129, *p* = 0.051), but no difference between the other leachate treatments and the control (black huckleberry, cattail, and autumn olive, *p* > 0.20 for all). Similarly, we found fewer moving zoospores as the leachate concentration increased (*X* ^2^(1) = 35.61, *p* < 0.001, *β* = −0.885 ± 0.167; Figure 1B). Leachate type also impacted the number of moving zoospores (*X* ^2^(6) = 64.52, *p* < 0.001); four of the leachate treatments decreased the number of moving zoospores compared to the control (native steeplebush [*β* = −1.058 ± 0.342, *p* = 0.002] and cattail [*β* = −0.856 ± 0.328, *p* = 0.009], invasive purple loosestrife [*β* = −1.750 ± 0.416, *p* < 0.001] and autumn olive [*β* = −1.867 ± 0.433, *p* < 0.001]) and two had no significant effect (native black huckleberry and invasive common reed; Figure 1B). Increasing the leachate concentration decreased the number of zoosporangia counted (*X* ^2^(1) = 303.72, *p* < 0.001, *β* = −0.761 ± 0.048; Figure 1C). Plant species also impacted zoosporangia counts; there was a significant decrease in the number of zoosporangia observed in all leachates compared to the control (*X* ^2^(6) = 161.76, *p* < 0.001, *β* estimates range from −1.01 to −0.22; Figure 1C). The interaction between leachate type and leachate concentration was not a significant predictor for any of the response variables.

### 3.2 In Vivo Experiment: Leachate Exposure on Tadpoles Before Infection

**FIGURE 2:**
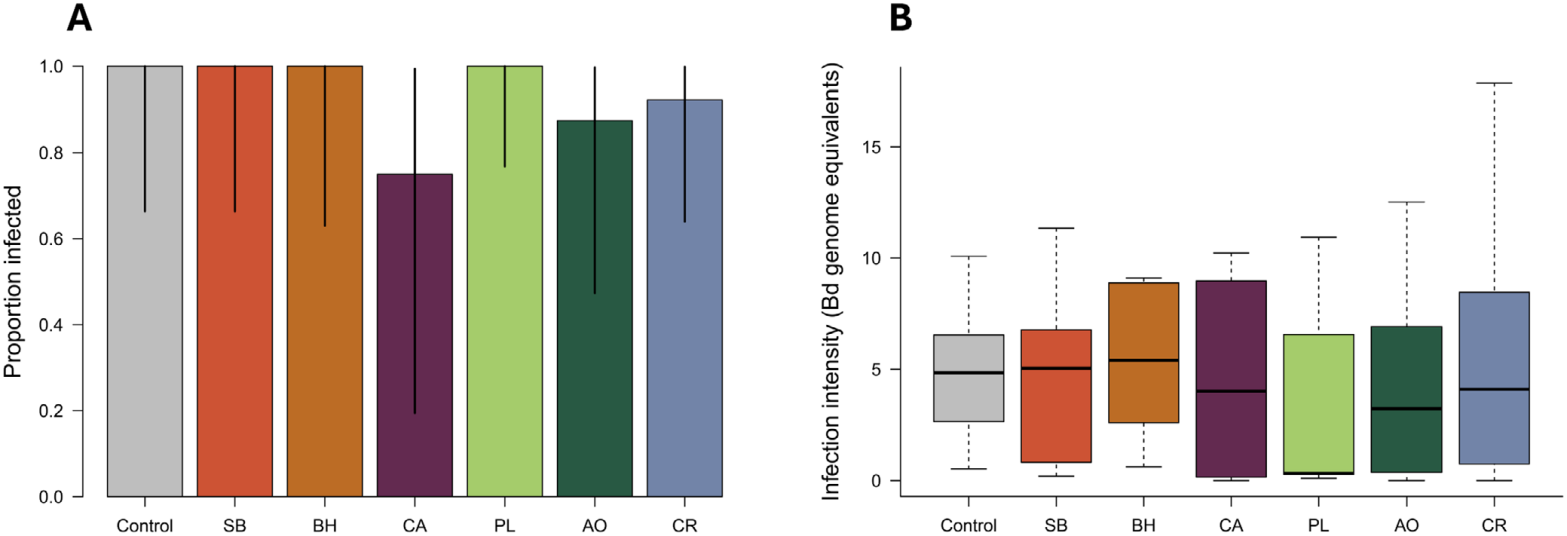
Results of the in vivo experiment: Leachate exposure on tadpoles before infection. The leachate types are represented as the native species SB (steeplebush), BH (black huckleberry), and CA (cattail), and the invasive species PL (purple loosestrife), AO (autumn olive), and CR (common reed). (A) The proportion of individuals infected at the end of the experiment is shown for each leachate type with 95% binomial confidence intervals. (B) The infection intensity is shown for each leachate type with boxplots showing the median (center line) and interquartile range (box) for each treatment and points representing outliers. There were no differences in infection prevalence or infection intensity across treatments in this experiment.

Across all treatments, 73.5% of individuals were infected in the in vivo experiment and there were no differences in infection prevalence (*X* ^2^(1) = 0.71, *p* = 0.399; Figure 2A), infection intensity (*X* ^2^(6) = 4.87, *p* = 0.561; Figure 2B), or survival (*X* ^2^(6) = 11.53, *p* = 0.073; Figure S3B) across leachate types. There were also no differences across treatments in mass or developmental stage at the end of the experiment (mass: *X* ^2^(7) = 9.39, *p* = 0.153; stage: *X* ^2^(1) = 0.22, *p* = 0.931; Figure S4). The average tadpole mass (g) at the end of the experiment was 0.058 (SD = ±0.025) and the average developmental stage was 25.05 (SD = 0.29).

## 4 Discussion

The movement of leaf litter from terrestrial systems into aquatic systems can have large effects on aquatic communities. In this study, we found that leachates can alter Bd growth in vitro (aim 1), but these effects did not translate to large impacts on infection when amphibian hosts were exposed to leachates prior to exposure (aim 2).

The effects of leachate on Bd in vitro varied by leachate type. For example, leachate from the invasive purple loosestrife affected all measures of Bd abundance, while leachate from black huckleberry only altered the number of zoosporangia (Figure 1). While the mechanism for these differences is unknown, previous studies suggest that chemical composition of leachates (Stoler, Burke, and Relyea 2016; DiGiacopo et al. 2019) and indirect effects mediated through the aquatic microbial community (Christian et al. 2017) may explain variation in the effects across leachate types. In our study, we sterilized the leachates, so we expect our results were primarily driven by differences in water chemistry and not variation in microbial communities across leachate types. For example, DiGiacopo et al. (2019) found that leachates from autumn olive, purple loosestrife, and common reed contained different levels of Mn, Ca, and K than those from swamp loosestrife, cattail, and huckleberry. In a follow‐up study, Meindl et al. (2020) manipulated the concentrations of metals to mimic the leachate chemical composition found in DiGiacopo et al. (2019) and found that shifts in chemical composition—especially increased Ca—affected amphibian survival. This underscores the important role chemical variation may play as a mechanism shaping the direct and indirect effects of leachates on disease outcomes. Additionally, there was no clear pattern indicating that leachate from invasive species had larger impacts on Bd than leachate from native species (Figures 1 and 2), despite previous studies predicting this result (Maerz et al. 2005; Burraco et al. 2018; Berta and Mott 2023). Thus, the chemical composition of leachates is likely a better predictor of its impacts on freshwater organisms than the origin of the plant species.

The different measures of Bd abundance in vitro responded differently to leachates. Our models indicated that higher concentrations of leachates reduced the abundance of all three measures of Bd: total number of zoospores, number of moving zoospores, and number of sporangia. These results are consistent with past studies, for example Stoler, Berven, and Raffel 2016, who found that when statistically pooled, increasing concentrations of leaf leachates from red maple, sugar maple, white oak, cottonwood, green ash, and phragmites resulted in reduced zoospore and sporangia concentrations. However, contrary to previous studies, when evaluating the effect of leachates from individual species, we found the total number of zoospores was actually higher in two leachate treatments (the invasive purple loosestrife and common reed) compared to the control indicating a potentially positive effect of leachates on zoospores. In contrast, four leachate treatments had lower abundance of moving zoospores, and all six leachate treatments had lower abundance of zoosporangia compared to the controls. The strong effect of leachates on zoosporangia may indicate that the zoosporangia stage of Bd is more susceptible to leachates than the zoospores stage. Overall, these findings suggest that shifts in leaf litter can affect pathogen growth, but the effects are not uniform across all plant species or invasion contexts. This reinforces the growing evidence that plant traits are essential for predicting the ecological impacts of litter inputs (Cohen et al. 2012; Stoler, Burke, and Relyea 2016).

Although we found that leachate can negatively affect Bd abundance in vitro, we found few impacts of leachates on infection in tadpoles (in vivo). Over our two tadpole‐exposure experiments (i.e., in vivo experiment and supplemental experiment) we only found one significant effect of leachates on infection where tadpoles in leachate from the invasive purple loosestrife had lower infection intensity than those from common reed in the supplemental experiment. Although leachates can have direct negative effects on amphibian hosts (Maerz et al. 2005; Watling et al. 2010; Earl and Semlitsch 2014; Burraco et al. 2018), we did not see any impact of leachates on survival, mass, developmental stage, or Bd infection in our in vivo experiment. The lack of effect could be due to the concentrations we used in our experiment; we chose to use a concentration of 1 g L^−1^ in our tadpole experiments to mimic concentrations commonly found in natural systems (Maerz et al. 2005; Stoler and Relyea 2013), but higher concentrations or a longer exposure period may be necessary to observe an effect on amphibian hosts. Additionally, the lack of effect on tadpole developmental stage is likely due to the long larval period of this species (Cook et al. 2013). In the field, indirect effects of leachate can be mediated through altered microbial communities and decreased dissolved oxygen (e.g., Zhao et al. 2021); processes that we expect had a minimal effect on tadpoles in our experiment due to frequent water changes. Future studies are necessary to identify the parameters by which leachates might impact tadpole infection with Bd in natural systems.

Leachate from purple loosestrife, an invasive species, had the highest number of significant effects on our response variables compared to any other leachate type. Purple loosestrife leachate affected all three measures of Bd abundance in the in vitro experiment (Figure 1) and was the leachate treatment with the lowest median infection in the supplemental experiment (Figure S2B). Similarly, a previous lab study evaluated the effects of leachates from autumn olive, huckleberry, swamp, and purple loosestrife on the susceptibility of American toads to trematodes and found that tadpoles exposed to purple loosestrife leachates had the highest effect on aquatic tadpoles, although this manifested as tadpoles in the purple loosestrife treatment having the highest level of trematode infection (DiGiacopo and Hua 2022). Therefore, purple loosestrife appears to have the largest negative effects on Bd compared to other leachate types and further investigation into the mechanisms by which purple loosestrife reduces Bd abundance and infection intensity in some scenarios may help identify additional species that have similar impacts.

We found no effect of purple loosestrife on amphibian mortality and growth (Figures S3 and S4). The lack of effect of purple loosestrife leachates on amphibians contrasts with some studies finding strong negative effects associated with purple loosestrife, including increased mortality and slowed growth of American toad tadpoles ( *Anaxyrus americanus* ; Maerz et al. 2005). Another study found that tadpoles exposed to purple loosestrife leachates had intermediate sizes and developmental rates compared to those exposed to leachates from other plants (swamp loosestrife, autumn olive, huckleberry; DiGiacopo and Hua 2022). These equivocal findings suggest that even within a plant species, the impacts of leachates vary across exposed aquatic species, space, and time, and underscore the importance of future investigation into the mechanisms by which plant traits shape disease outcomes as opposed to plant identity alone.

Our results indicate that leachates can have direct negative effects on aquatic organisms, but these effects do not always translate into changes in host‐pathogen interactions. Our study included several simplifications that are unlikely to occur in natural systems including a constant leachate concentration through time, the use of only one leaf litter species at a time (i.e., no mixed leachate type treatments), and the removal of potential indirect effects of leachates mediated through shifts in the microbial communities. Additionally, in natural systems, interactions between terrestrial plants, pathogens, and aquatic hosts could lead to unexpected impacts on host‐pathogen interactions. For example, a previous study found that higher tree canopy cover was associated with higher Bd infection due to the cooling effect of the canopy creating favorable conditions for the pathogen (Becker et al. 2012). Thus, future studies should build upon our work to consider the complex interactions between leaf litter and aquatic organisms that occur in natural systems. However, our findings have applications for the general understanding of how leaf litter affects aquatic organisms, and particularly aquatic host‐pathogen interactions. Overall, understanding the effects of leachates on freshwater systems will allow for a better understanding of the general connections between terrestrial and aquatic ecosystems.

## Author Contributions

**Emily L. Martin:** conceptualization (equal), investigation (equal), methodology (equal), visualization (equal), writing – original draft (equal), writing – review and editing (equal). **Isabel Adarve‐Rengifo:** data curation (equal), investigation (equal), methodology (equal), writing – review and editing (equal). **Spencer R. Siddons:** conceptualization (equal), investigation (equal), methodology (equal). **Paradyse E. Blackwood:** investigation (equal), methodology (equal), writing – review and editing (equal). **Jessica Hua:** methodology (equal), resources (equal), writing – review and editing (equal). **Catherine L. Searle:** conceptualization (equal), formal analysis (equal), funding acquisition (equal), project administration (equal), resources (equal), supervision (equal), visualization (equal), writing – original draft (equal), writing – review and editing (equal).

## Funding

This work was supported by Directorate for Biological Sciences, DEB 2044897, DEB 2314625.

## Conflicts of Interest

The authors declare no conflicts of interest.

## Supporting information


**Figure S1:** Pictures of the soaking process for creating leachates. Leachates were created by soaking dry leaf litter in 50 L cattle tanks for 40 days and then filtered through 153 μm mesh to remove large particles. Initial leaf litter concentrations were 10 g L‐1 for the in vitro experiment and 1 g L‐1 for the in vivo experiment (dry mass). Control treatments only contained clean well water.
**Table S1:** Summary of replicate numbers for the in vitro experiment and supplemental experiment. The leachate types are represented as the native species SB (steeplebush), BH (black huckleberry), and CA (cattail), and the invasive species PL (purple loosestrife), AO (autumn olive), and CR (common reed). Numbers in parentheses are the number of tadpoles that survived until the end of the experiment in each treatment. The supplemental treatment did not include a water control and is therefore indicated with “N/A” for “not applicable.”
**Figure S2:** Infection results from the supplemental experiment: Continuous tadpole leachate exposure. The leachate types are represented as the native species SB (steeplebush), BH (black huckleberry), and CA (cattail), and the invasive species PL (purple loosestrife), AO (autumn olive), and CR (common reed). (A) The proportion of individuals infected at the end of the experiment is shown for each leachate type with 95% binomial confidence intervals. (B) The infection intensity is shown for each leachate type with boxplots showing the median (center line) and interquartile range (box) for each treatment and circles representing outliers. Infection prevalence did not differ across treatments, but the common reed (CR) treatment group had higher infection intensity than purple loosestrife (PL).
**Table S2:** Average water quality measurements from the six leachate treatments used in experiment #2.
**Figure S3:** Survival in (A) the supplemental experiment and (B) the in vivo experiment was not statistically different across treatments. The leachate types are represented as the native species SB (steeplebush), BH (black huckleberry), and CA (cattail), and the invasive species PL (purple loosestrife), AO (autumn olive), and CR (common reed).
**Figure S4:** (A) Mass and (B) developmental stage of tadpoles from the in vivo experiment that survived until the end of the experiment. The leachate types are represented as the native species SB (steeplebush), BH (black huckleberry), and CA (cattail), and the invasive species PL (purple loosestrife), AO (autumn olive), and CR (common reed). Boxplots show the median (center line) and interquartile range (box) for each treatment with circles representing outliers.

## Data Availability

The data associated with this manuscript are publicly available on Github (github.com/SearleLabData/Leachate) and we will archive it on Dryad (https://doi.org/10.5061/dryad.t4b8gtjhq) upon acceptance.
