# Supplementary material for: Leaf Litter Leachate Limits Fungal Pathogen Growth but Not Amphibian Infection
Source: Ecol Evol. 2026 May 13;16(5):e73668. doi: 10.1002/ece3.73668 (PMC13171222; doi:10.1002/ece3.73668)
Supplement: Supplementary file 1 — Figure S1: Pictures of the soaking process for creating leachates. Leachates were created by soaking dry leaf litter in 50 L cattle tanks for 40 days and then filtered through 153 μm mesh to remove large particles. Initial leaf litter concentrations were 10 g L‐1 for the in vitro experiment and 1 g L‐1 for the in vivo experiment (dry mass). Control treatments only contained clean well water. Table S1: Summary of replicate numbers for the in vitro experiment and supplemental experiment. The leachate types are represented as the native species SB (steeplebush), BH (black huckleberry), and CA (cattail), and the invasive species PL (purple loosestrife), AO (autumn olive), and CR (common reed). Numbers in parentheses are the number of tadpoles that survived until the end of the experiment in each treatment. The supplemental treatment did not include a water control and is therefore indicated with “N/A” for “not applicable.” Figure S2: Infection results from the supplemental experiment: Continuous tadpole leachate exposure. The leachate types are represented as the native species SB (steeplebush), BH (black huckleberry), and CA (cattail), and the invasive species PL (purple loosestrife), AO (autumn olive), and CR (common reed). (A) The proportion of individuals infected at the end of the experiment is shown for each leachate type with 95% binomial confidence intervals. (B) The infection intensity is shown for each leachate type with boxplots showing the median (center line) and interquartile range (box) for each treatment and circles representing outliers. Infection prevalence did not differ across treatments, but the common reed (CR) treatment group had higher infection intensity than purple loosestrife (PL). Table S2: Average water quality measurements from the six leachate treatments used in experiment #2. Figure S3: Survival in (A) the supplemental experiment and (B) the in vivo experiment was not statistically different across treatments. The leachate types are repre [file ECE3-16-e73668-s001.pdf]

Supplemental Information for:
Leaf litter leachate limits fungal pathogen growth but not amphibian infection

Emily L. Martin, Isabel Adarve-Rengifo, Spencer R. Siddons, Paradyse E. Blackwood, Jessica Hua, and Catherine L. Searle

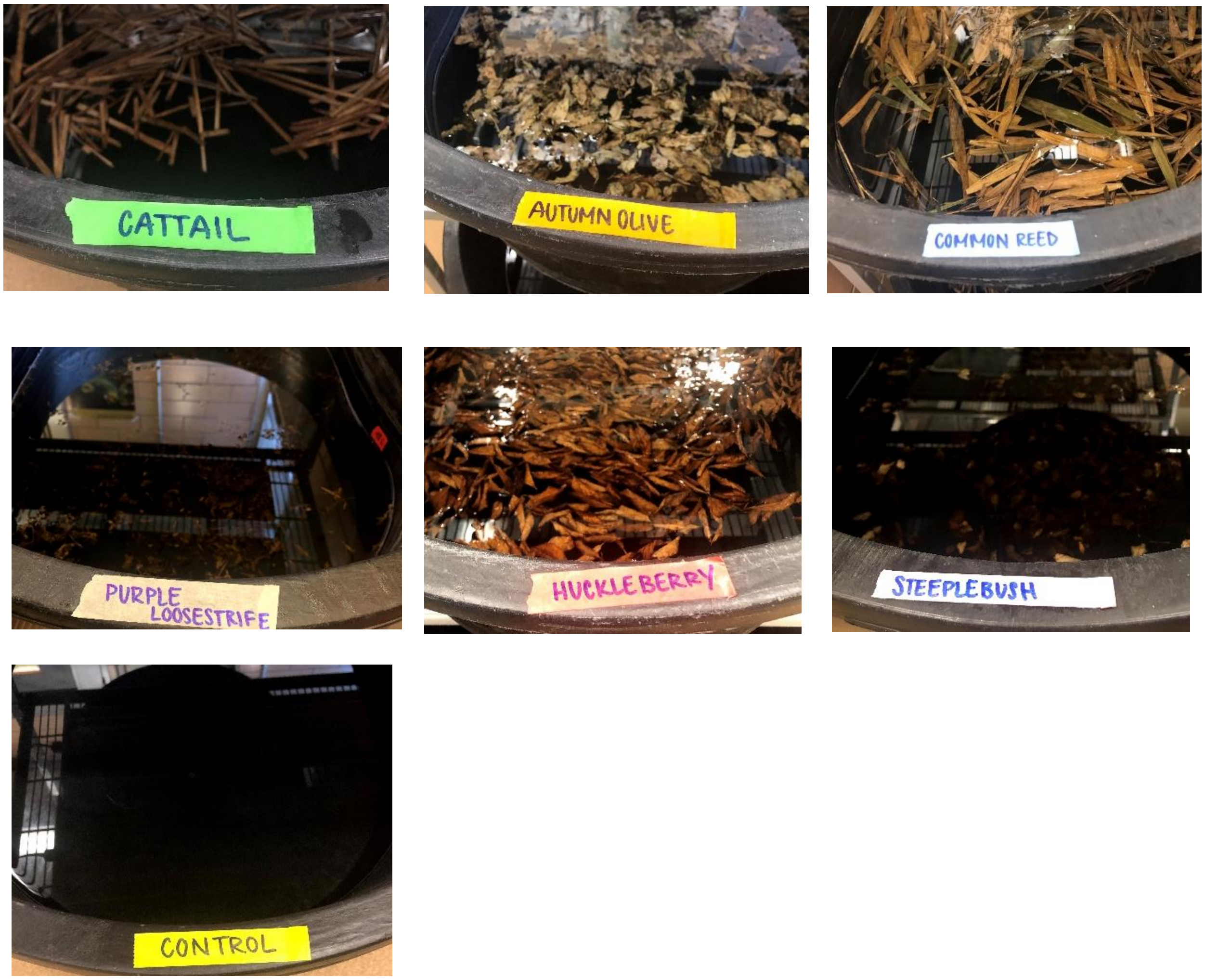

Figure S1: Pictures of the soaking process for creating leachates. Leachates were created by soaking dry leaf litter in 50 L cattle tanks for 40 days and then filtered through 153µm mesh to remove large particles. Initial leaf litter concentrations were 10 g L^{-1} for the *in vitro* experiment and 1 g L^{-1} for the *in vivo* experiment (dry mass). Control treatments only contained clean well water.

Table S1: Summary of replicate numbers for the *in vitro* experiment and supplemental experiment. The leachate types are represented as the native species SB (steeplebush), BH (black huckleberry), and CA (cattail), and the invasive species PL (purple loosestrife), AO (autumn olive), and CR (common reed). Numbers in parentheses are the number of tadpoles that survived until the end of the experiment in each treatment. The supplemental treatment did not include a water control and is therefore indicated with "N/A" for "not applicable."

Treatment	*In vivo* experiment number of replicates (number survived to end)	Supplemental experiment number of replicates (number survived to end)	Supplemental experiment number of replicates missing infection data
SB	20 (9)	31(25)	1
BH	20 (8)	30 (23)	1
CA	18 (4)	31 (27)	6
PL	19 (14)	32 (23)	0
AO	20 (8)	35 (31)	1
CR	19 (13)	32 (28)	5
Water	19 (9)	N/A	N/A

Supplemental experiment: Continuous tadpole leachate exposure

Methods

The supplemental experiment tested the effects of continuous exposure of the tadpoles to leachates before, during, and after Bd exposure. We had 6 leachate treatments (one for each leaf litter type), at a concentration of 1g L^{-1}. Each treatment was replicated 30-35 times (Table S1) and each replicate was a single tadpole in 50mL of leachate in a 400mL glass beaker (192 total experimental units). The relatively low volume of water was used to ensure a high concentration of Bd zoospores. We weighed 30 extra animals on day 1 to estimate starting mass. At the beginning of the experiment, tadpoles were placed into the beakers for 24 hours before Bd inoculation. Beakers were inoculated with Bd to create a concentration of 2.0×10^5 zoospores mL^{-1} in each beaker. The Bd concentration was different from the *in vivo* experiment in the main text due to availability of Bd zoospores. Tadpoles remained in the Bd-exposed water for 10 hours after which they were moved to new beakers with 250mL of their assigned leachate treatment without Bd for an additional 6 days. Water quality measurements from the leachates in this experiment are shown in Table S2.

Beakers were randomly assigned spatially to one of 4 shelves in the laboratory. Tadpoles were checked daily for mortality. On the final day of the experiment, all individuals were euthanized and preserved in ≥90% ethanol until processing for infection analysis, which followed methods outlined in the *in vitro* experiment in the main text. Due to loss of samples, we were unable to verify infection status for 14 individuals that survived (Supplemental table S1).

Results

Infection prevalence was high across treatments (93.9% infected across the whole experiment) and leachate type did not significantly affect infection prevalence ($X^2(1) = 0.147$, $p = 0.702$; Figure 2A). Infection intensity differed across leachate types ($X^2(5) = 15.85$, $p = 0.007$); post-hoc tests revealed only one significant pairwise comparison where the invasive common reed treatment group had higher infection intensity than invasive purple loosestrife ($p = 0.022$); Figure 2B). Survival was high (83.0% survival across treatments) and not affected by leachate type ($X^2(5) = 4.35$, $p = 0.500$; Figure S3A). The average starting mass of the tadpoles was 0.017g (SD = ±0.005).

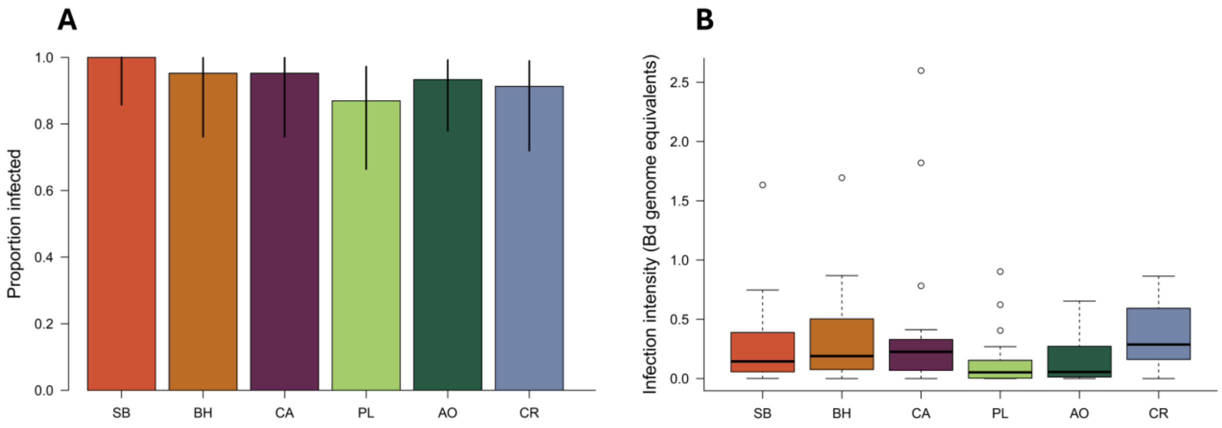

Figure S2: Infection results from the supplemental experiment: Continuous tadpole leachate exposure. The leachate types are represented as the native species SB (steeplebush), BH (black huckleberry), and CA (cattail), and the invasive species PL (purple loosestrife), AO (autumn olive), and CR (common reed). A) The proportion of individuals infected at the end of the experiment is shown for each leachate type with 95% binomial confidence intervals. B) The infection intensity is shown for each leachate type with boxplots showing the median (center line) and interquartile range (box) for each treatment and circles representing outliers. Infection prevalence did not differ across treatments, but the common reed (CR) treatment group had higher infection intensity than purple loosestrife (PL).

Table S2: Average water quality measurements from the six leachate treatments used in experiment #2.

Species (abbreviation)	Invasive status	pH	Salinity (ppt)	DO (mg/L)	Turbidity
Steeplebush (SB)	Native	7.74	0.43	1.82	405
Black huckleberry (HB)	Native	7.89	0.30	4.27	303
Cattail (CA)	Native	8.11	0.72	1.50	141
Purple loosestrife (PL)	Invasive	7.50	0.66	2.27	269
Autumn olive (AO)	Invasive	8.11	0.54	2.30	220
Common reed (CR)	Invasive	8.40	0.67	2.15	27

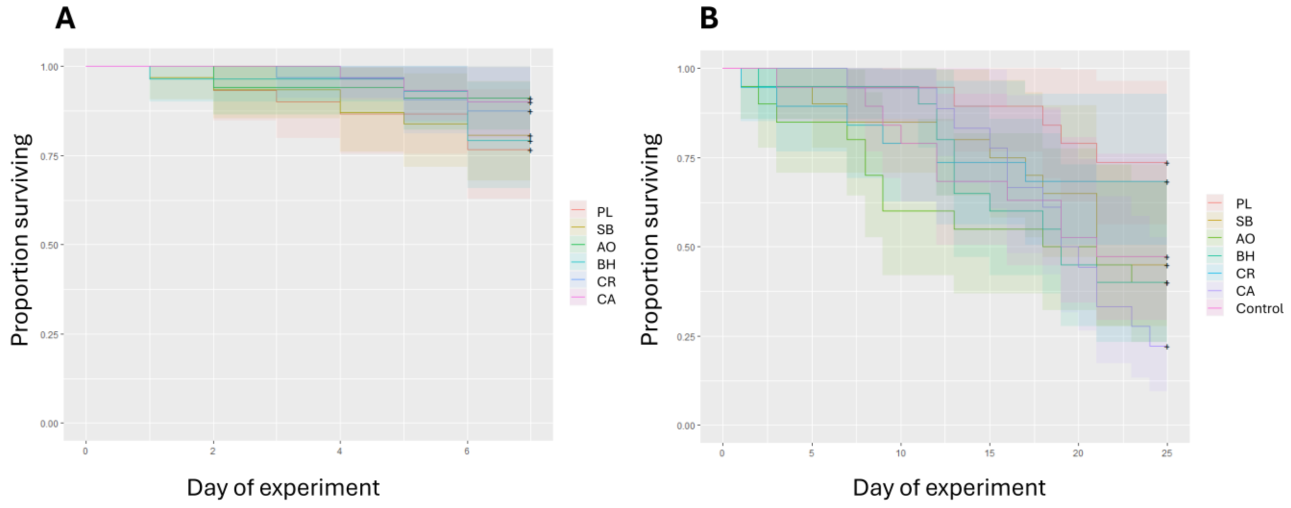

Figure S3: Survival in A) the supplemental experiment and B) the *in vivo* experiment was not statistically different across treatments. The leachate types are represented as the native species SB (steeplebush), BH (black huckleberry), and CA (cattail), and the invasive species PL (purple loosestrife), AO (autumn olive), and CR (common reed).

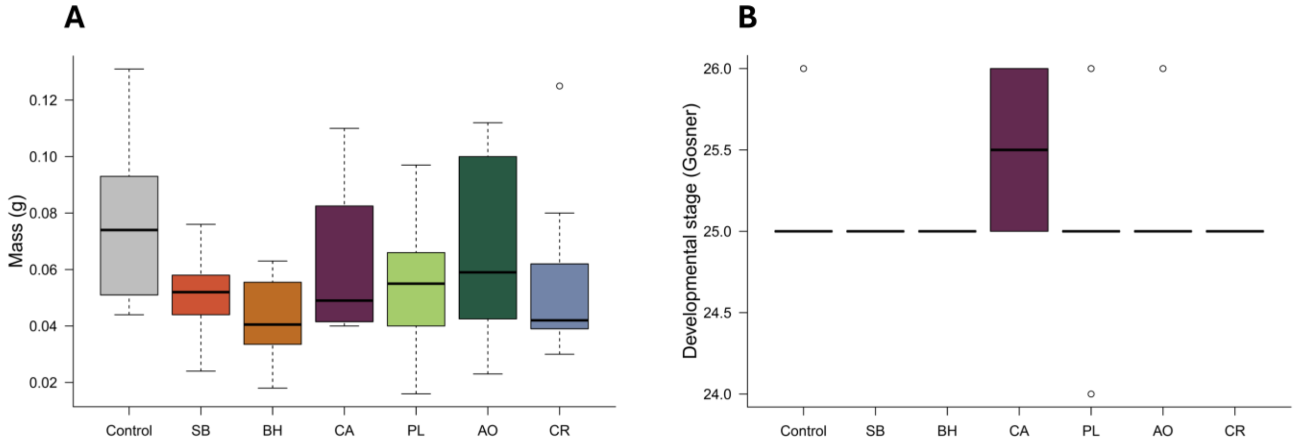

Figure S4: A) Mass and B) developmental stage of tadpoles from the *in vivo* experiment that survived until the end of the experiment. The leachate types are represented as the native species SB (steeplebush), BH (black huckleberry), and CA (cattail), and the invasive species PL (purple loosestrife), AO (autumn olive), and CR (common reed). Boxplots show the median (center line) and interquartile range (box) for each treatment with circles representing outliers.
